# Proteomic Studies of Blood and Vascular Wall in Atherosclerosis

**DOI:** 10.3390/ijms222413267

**Published:** 2021-12-09

**Authors:** Ekaterina Mikhailovna Stakhneva, Evgeniia Vitalievna Striukova, Yulia Igorevna Ragino

**Affiliations:** The Institute of Internal and Preventive Medicine, Branch of the Federal Research Center Institute of Cytology and Genetics, Siberian Branch of Russian Academy of Sciences, St. B. Bogatkova 175/1, 630089 Novosibirsk, Russia; stahneva@yandex.ru (E.M.S.); ragino@mail.ru (Y.I.R.)

**Keywords:** proteomics, atherosclerosis, biomarkers, mass spectrometry

## Abstract

The review is devoted to the analysis of literature data related to the role of proteomic studies in the study of atherosclerotic cardiovascular diseases. Diagnosis of patients with atherosclerotic plaques before clinical manifestations is an arduous task. The review presents the results of research on the new proteomic potential biomarkers of coronary heart disease, coronary atherosclerosis, acute coronary syndrome, myocardial infarction, carotid artery atherosclerosis. Also, the analysis of literature data on proteomic studies of the vascular wall was carried out. To assess the involvement of proteins in the pathological process of atherosclerosis, it is important to investigate the specific relationships between proteins in the arteries, expression and concentration of proteins. The development of proteomic technologies has made it possible to analyse the number of proteins associated with the development of the disease. Analysis of the proteomic profile of the vascular wall in atherosclerosis can help to detect possible diagnostically significant protein structures or potential biomarkers of the disease and develop novel approaches to the diagnosis of atherosclerosis and its complications.

## 1. Introduction

One of the most promising areas of molecular biology is proteomics, whose tasks include the study of gene expression protein products, including their posttranslational modifications and their comparative analysis. Proteomic studies are a systematic study of all proteins of the body, the study of the involvement of each protein in various physiological and pathological processes, the determination of the potential use of proteins as effective diagnostic markers. As a rule, proteomic studies are carried out using a combined use of methods: two-dimensional electrophoresis (2D electrophoresis), mass spectrometric analysis of the molecular weight and sequence of proteins separated by electrophoresis of biological material, followed by the analysis of the results obtained by bioinformatics methods.

The intensive development of mass spectrometric analysis has contributed to the emergence in recent years of a whole group of areas of proteomic research. Using a combination of these methods, it is possible to create a proteomic map of biological material, which is a phenotypic manifestation of the genome of a cell, tissue, or even an entire organ. Proteomic analysis can be used not only for the inventory of specific biological object proteins but also for the control of reversible posttranslational modifications of proteins by specific enzymes, such as phosphorylation, glycosylation, acylation, etc. Thus, proteomic profiling is successfully used to search for specific sets of proteins for various tissues in pathology.

Blood proteins are the most promising sources for the biomarker search. To date, more than 10 thousand proteins have been identified in blood plasma (http://www.plasmaproteomedatabase.org, 7 December 2021). However, the range of concentrations is extensive: from femtomolar concentrations to major fractions of proteins, such as serum albumin and immunoglobulins, which make up about 99% of the total amount of protein in the blood. In addition, blood proteins are very variable due to posttranslational modifications.

Cardiovascular diseases are considered the main causes of morbidity and mortality worldwide. From uncovering the aetiology of the disease to determining prognostic markers for better disease management, there are still many unresolved questions for researchers. In the era of human genome sequencing, most of the researches were focused on the use of advanced genomic tools along with the assessment of traditional risk factors. With the development of next-generation proteomics and next-generation metabolism approaches, it has become possible to understand the protein interaction network and metabolic rearrangement that lead to disorders of the disease phenotype. In recent years, many studies have been conducted in which proteomics has been successfully applied to identify potential prognostic and diagnostic markers, as well as to understand the mechanisms of the disease for various types of CVD. The detection and verification of new protein biomarkers of CVD, in particular, coronary artery atherosclerosis, is a very difficult task. Even though the number of studied proteins—potential prognostic and diagnostic biomarkers is constantly growing, their role in the pathogenesis of coronary atherosclerosis is not always clear. It is necessary to understand the changes in the protein component of the blood at an early stage of atherosclerosis, which will help to identify new candidate biomarkers for detecting this disease before the appearance of clinical manifestations. Therefore, it is highly important to choose a method of proteomic analysis that allows overcoming the difficulties associated with a wide concentration range of proteins present and their posttranslational modifications.

Identification of proteins by mass spectra using databases is time-consuming and requires the high professionalism of the operator. Given that proteins are characterized by a wide range of isoforms, including posttranslational modifications and mutations, it is not always possible to accurately identify the protein because protein isoforms are not included in proteomic databases. Often, multiple protein isoforms are identified as single proteins. An important task of biological and medical research is the identification of a specific protein isoform [1,2]. Therefore, methods of mass spectrometry and peptide identification methods are constantly being improved.

Multicentre projects were carried out to evaluate and control the quality of proteomic studies conducted. Thus, a large-scale multicentre study was conducted to quantify the measurements of human plasma proteins multiple reaction monitoring based on the mass spectrometry method. Reproducibility was evaluated, the linear dynamic range was determined, detection limits were determined, and multiplexed proteomic assays were quantified. The high reproducibility and sensitivity of proteomic analyses in blood plasma within each of the Laboratories participating in the multicentre study were determined. Data and benchmarks were provided, according to which individual Laboratories can compare their effectiveness and evaluate new technologies for testing proteomic biomarkers in blood plasma [3,4].

Scientific research works on proteomics are designed/intended to improve the diagnosis, prognosis and treatment of diseases in clinical practice. Proteomic studies have made it possible to include in guidelines natriuretic peptide B-type (BNP) and/or N-terminal prohormone natriuretic peptide B-type (NT-proBNP) for the diagnosis of heart failure and prognostic evaluation of patients with chronic heart failure [5,6]. Natriuretic peptides and cardiac troponins are established blood biomarkers in the diagnosis of heart failure and the prognosis of outcomes associated with heart failure [7]. Thanks to non-invasive determination with high sensitivity and accuracy, proteomic biomarkers of circulating blood are becoming increasingly important for everyday clinical practice.

This review presents the results of proteomics-based studies that are important for identifying potential biomarkers for the early diagnosis or prognosis of atherosclerotic CVD, for elucidating new features of the mechanisms, for understanding the pathophysiology of CVD, primarily coronary atherosclerosis.

## 2. Results of Proteomic Blood Tests in Population Groups of Individuals and Non-Complicated Coronary Heart Disease

Thanks to proteomic technologies, it has become possible to discover new biomarker proteins, each of which is evaluated individually as prognostic clinical data. Assessments of the protein complex are proposed as a promising approach for more accurate diagnosis and choice of treatment tactics to prevent the development of disease complications.

Attempts have been made to determine the spatial distribution of the human proteome at the subcellular level to improve the understanding of human biology and diseases. In situ mapping of the 12,003 human proteins localization at the unicellular level in 30 subcellular structures made it possible to determine the proteomes of 13 main organelles [8].

Despite the existence of several models for stratifying the risk of developing cardiovascular complications, studies were conducted to optimize existing models and discover new protein panels that can stratify patients throughout their lives [9]. Proteomic biomarkers associated with risk factors for cardiovascular diseases can provide insight into the pathogenesis of cardiovascular diseases. The analysis of 1305 proteomic biomarkers of blood revealed statistically significant associations with factors: 60 proteins with smoking, 30 proteins with alcohol consumption and five proteins with physical activity. The loci of quantitative signs of the protein were associated with coronary heart disease, inflammation and age-related mortality [10].

In a prospective cohort study in patients with coronary heart disease (CHD) (cohort # 1: San Francisco, 11.1 years of prospection; cohort # 2: Norway, 5.6 years of prospection), 1130 proteins were studied in blood plasma using proteomic analysis. From cohort No. 1, 938 samples were analysed, the average age was 67.0 years, and 82% were men. 971 samples from cohort No. 2 were analysed, the average age was 70.2 years, and 72% were men. Two risk assessment systems (a complex of 9 proteomic analysis proteins versus a Framingham riskometer) were compared concerning the predictive assessment of developing CHD complications risk during five years of follow-up. In patients with CHD, the assessment of the myocardial infarction, stroke, heart failure and death from all causes risk in 5 years using a complex of 9 proteomic analysis proteins showed better results than the Framingham riskometer. The authors concluded that further studies are needed to assess the risk of developing these complications using a complex of 9 proteins in the population not only with CHD but also without it [11].

An analysis of the systemic biology of changes in high-density lipoprotein (HDL) protein in blood serum caused by percutaneous transluminal coronary angioplasty (PTCA) in patients with CHD revealed an increase in apolipoprotein proteins, fibrinogen-like protein 1, as well as a decrease in antithrombin-III, annexin A1 and several immunoglobulins [12].

In the study of the effect of myeloperoxidase (MP) on the function of high-density lipoprotein cholesterol (HDL-C) and on HDL-proteome in 4 groups of ethnic Chinese (20 patients per group): (1) persons with a low level of HDL-C, without CHD; (2) persons with a low level of HDL-C and with CHD; (3) persons with a high level of HDL-C, without CHD; (4) persons with a high level of HDL-C with CHD. The cytokine proteome, MP, MP-oxidized tyrosine fragments in blood serum and the HDL proteome were evaluated individually by mass spectrometry in 4 groups. The authors concluded that MP-mediated oxidative stress and changes in the composition of HDL-C are not the main cause of HDL particle dysfunction in Chinese subjects with CHD. The results of the study reflect ethnic differences in HDL dysfunction between the cohorts of China and the United States, where opposite results were obtained [13].

When diagnosing the early stage of the disease, special attention is paid to acute-phase proteins. Using proteomic analysis, 298 proteins associated with the pathogenesis of coronary atherosclerosis were specifically studied in 63 people (33 with coronary heart disease and 30 in the control group). 82 differentially expressed proteins were identified that distinguish coronary atherosclerosis, including C-reactive protein, vascular endothelial growth factor, NO synthase and adhesive molecules of vascular endothelium type 1 [14].

Combined proteomic and glycomic profiling of HDL particles in 10 patients with coronary angiographically verified coronary atherosclerosis and 10 control group individuals revealed a lower blood content of HDL apolipoproteins (in particular, ApoA-I, ApoA-II and apoE), serum amyloid A2 (SAA2), but a higher content of sialylated glycans in patients with coronary heart disease. The authors concluded that the combined proteomic and glycomic profiling of HDL particles was tested as a new analytical approach for the development of new potential biomarkers of coronary atherosclerosis [15].

Ceruloplasmin-a specific copper-containing plasma glycoprotein belongs to the acute phase proteins. The synthesis of ceruloplasmin is carried out by hepatocytes. In the body, the protein performs several important biological functions: it increases the stability of all cell membranes, participates in the reactions of the immune response, prevents lipid peroxidation, inactivates oxygen free radicals, stimulates haematopoiesis, participates in the reactions of iron addition to haemoglobin [16]. Proteomic research methods have shown that in patients with coronary heart disease and heart failure, a reduced level of ceruloplasmin is an unfavourable prognostic sign [17]. In patients with coronary atherosclerosis, a decrease in the level of one isoform and two fragments of ceruloplasmin in the blood serum was identified [18]. And in other studies, heart failure is associated with high levels of ceruloplasmin [19]. When studying the associations of the genetic variants concentration of ceruloplasmin and the occurrence of atrial fibrillation, it was shown that these variants are associated with an increased concentration of ceruloplasmin in the blood, but at the same time reduce the risk of fibrillation [20].

For the early diagnosis of the disease, special attention is drawn to proteins involved in the implementation of the immune response of the body, proteins of the complement system. Many studies show the activation of the complement system in atherosclerosis [21,22,23]. In addition, the level of C4 protein mRNA is known to be significantly increased in atherosclerotic plaque [24]. Proteomic studies of the blood of patients with coronary atherosclerosis showed an increase in the content of components of the complement system C3 (chain B), C4, C9 and a decrease in the level of the complement component C3 (chain C) [18].

Vitronectin is one of the main protein components of blood plasma, participates in fibrinolysis, inhibits the membrane-attacking cytolytic complex of the complement system. Using proteomic research methods, 2D electrophoresis and mass spectrometric analysis, increased concentrations of the complement system sub-components C1r, C3 and C4-B, α-1-antitrypsin and vitronectin were detected in patients with type 2 diabetes mellitus compared to the control group. These data suggested that vitronectin play an important role in the development of diabetic atherosclerosis [25].

The proprotein convertase of subtilisin-kexin type 9 (PCSK9) belongs to the family of proprotein convertases that activate enzymes by cleaving off a peptide that inhibits their catalytic activity. PCSK9 plays an important regulatory role in the homeostasis of TC. The binding of PCSK9 to the EGF-A domain of the low-density lipoprotein (LDL) receptor leads to the degradation of the receptor. A decrease in the level of the LDL receptor, in turn, causes a reduced LDL metabolism, which can lead to hypercholesterolemia [26]. The blood serum of 334 patients was analysed using the Proximity Extension Assay technology. Associations were found between the development of aortic valve stenosis in patients with coronary atherosclerosis and certain proteins, such as differential growth factor-15, galectin-4, the Willebrand factor, interleukin 17, transferrin receptor protein 1 and PCSK9 [27].

Galectin-3 belongs to the family of β-galactoside-binding proteins, plays an important role in cell adhesion, macrophage activation, angiogenesis, and apoptosis. Galectin-3 enhances pro-inflammatory signals, has chemotactic properties in relation to macrophages and monocytes, induces neutrophil adhesion, participates in neutrophil phagocytosis by macrophages. In the Langley study, when studying proteins-potential biomarkers of atherosclerosis progression, four proteins (matrix metalloproteinase 9, calprotectin, cathepsin D and galectin-3) were identified, which made it possible to improve the prediction of the risk of complications of the atherosclerotic process [28].

Lind and colleagues used extended proteomic analysis (Proseek Multiplex CVD, Olink Bioscience, Uppsala, Sweden) to study 82 plasma proteins associated with atherosclerotic cardiovascular diseases in a population of elderly people (931 people, 50% of men and 50% of women aged 70 years) to identify the connection of these proteins with the spread of plaques in the carotid arteries. Seven proteins were significantly associated with the number of atherosclerotic plaques in the carotid arteries (osteoprotegerin, T-cell immunoglobulin and mucin domain 1, growth differentiation factor 15, matrix metalloproteinase-12, renin, a member of the tumour necrosis factor superfamily 14 (TNFSF14) and growth hormone). Of these, renin (odds ratio [OR], 1.30, confidence interval 95% [CI], 1.13–1.49 per standard deviation), growth hormone (OR, 1.24, 95% CI, 1.08–1.43), osteoprotegerin (OR, 1.22, 95% CI, 1.05–1.43) and TNFSF14 (OR, 1.17, 95% CI, 1.01–1.35) were associated with the spread of plaques independently of each other and from traditional CVD risk factors [29].

When studying the relationship between serum sphingolipids and CHD using the method of liquid chromatography with tandem mass spectrometry, it was found that almost every measured sphingolipid (30 out of 32) was significantly increased in subjects with CHD compared to measurements in the control population. The authors created a new CHD risk scale (SIC), including sphingolipids, which distinguishes patients with CHD independently and more effectively than conventional clinical biomarkers of CVD, including LDL cholesterol and serum triglycerides [30].

And increased concentrations of renin and growth differentiation factor 15 (GDF15) proteins and a lower adiponectin level were independently associated with coronary heart disease and diabetes mellitus [31]. In a proteomic study of Monu with co-authors, differences in 19 proteins were identified in the blood of patients with coronary heart disease compared to healthy ones. Serotransferrin, talin-1, glycoprotein alpha-2HS and transthyretin were found to be lower, while fibrinogen α-chain to be higher in the plasma of CHD. The data of mass spectrometric analysis were confirmed by Western blot analysis. Low levels of transthyretin, an important transport protein of the acute phase, were confirmed in all patients with coronary heart disease. The authors suggested that a lower level of transthyretin allows predicting the severity of the disease and can serve as an important marker for the screening of coronary heart disease [32]. But in an earlier study, an increase in the content of transthyretin, hemopexin and retinol-binding protein-4 was found in blood serum samples of patients with verified coronary atherosclerosis. At the same time, an increase in the content of the monomeric form of transthyretin is associated with atherosclerosis [18].

Two mass spectrometric approaches were used in the study of blood proteomic profile dependence on the severity of coronary artery atherosclerosis in patients with diabetes and varying degrees of coronary artery stenosis: (1) tandem mass spectrometry with traditional liquid chromatography for detection; (2) quantitative MRM for verification and confirmation. The analysis showed that apolipoprotein C-II (APOC2), dedicator of cytokinesis protein 2 (DOCK2), ligand 7 (CXCL7) and vitamin D binding protein (VTDB) were activated, and complement 4A (C4A) were suppressed in diabetic patients and were associated with severe coronary artery stenosis. Human lipopolysaccharide-binding protein (LBP) and vitamin D binding protein (VTDB) were suppressed in patients without diabetes and were associated with severe coronary artery stenosis [33].

## 3. Results of Proteomic Blood Tests in Acute Coronary Syndrome and Myocardial Infarction

The development of proteomic technologies has made it possible to analyse the number of proteins associated with the development of the disease. These changes reflect the molecular and cellular mechanisms and can make it possible to predict the dynamics of the disease.

Endothelial dysfunction is one of the main factors of the occurrence and progression of atherothrombosis, leading to acute myocardial infarction (AMI). To better understand the endothelial proteome and the pathophysiology of AMI, protein profiles in patients with AMI were studied using proteomic methods. 2246 proteins were identified and quantified, of which 335 were differentially regulated in endothelial cells obtained from atherothrombotic material of the occluded coronary artery in patients with AMI. Differentially regulated proteins show changes in RNA metabolism, changes in platelet activation, signal transmission and aggregation, neutrophil degranulation, amino acid and derivative metabolism, changes in cellular responses to stress [34].

Using differential Q-proteomics, the proteins of serum extracellular vesicles were studied in 471 patients with the suspected acute coronary syndrome (ACS) compared to the control group of individuals. Significant independent associations of polygenic immunoglobulin receptor, cystatin C and complement factor C5a with ACS were revealed, especially in men. The authors concluded that these observations should be confirmed in a large prospective study to assess the potential role of these proteins in the ACS risk of developing [35].

Using mass spectrometry, 861 plasma proteins were studied in 135 people with myocardial infarction and 135 people of the control group. A complex of 7 proteins (cyclophilin A, an antigen-like cluster of differentiation 5 (CD5), cell-surface glycoprotein mucin cell surface-associated protein 18 (MUC-18), collagen chain-1, salivary α-amylase 1, C-reactive protein and multimerin-2) was determined, independently associated with myocardial infarction and with its more favourable prognosis. A complex of 4 proteins (α-1-acid glycoprotein 1, paraoxonase 1, tetranectin and CD5) associated with the risk of developing ACS was also identified. The authors concluded that the results of their research contribute to improving the early diagnosis of ACS and contribute to a better understanding of the mechanisms of its development [36].

The plasma proteome of 30 patients with acute myocardial infarction (AMI) was analysed to search for new prognostic biomarkers. Proteomic analysis of blood samples was performed using 2D electrophoresis after purification from albumin and immunoglobulin G. It was found that a low level of haptoglobin and its isoforms in blood plasma, as well as the presence of its α2-α2 genotype, are associated with a more severe class of heart failure and a negative prognosis of the myocardial infarction course. The level of haptoglobin in plasma below 1.4 g/L predicted the occurrence of heart failure (according to NYHA 2, 3, 4) for one year with 100% sensitivity [37].

But the current study does not confirm these results. When studying blood biomarkers in young people (18–45 years old) with AMI, it was found that the concentration of haptoglobin in the blood was increased and independently associated with AMI. The study was conducted using mass spectrometry and verification using an enzyme immunoassay in the blood serum of patients with AMI compared to a healthy control group. A positive correlation with a highly sensitive C-reactive protein (r = 0.424, *p* < 0.001) suggests that haptoglobin may be a potential biomarker of AMI in young people [38].

Heart failure is the most common complication of AMI in the long-term prognosis. Using proteomic analysis, Raizada and colleagues found biomarkers associated with heart failure in ACS—natriuretic peptide B-type, differential growth factor-15, a protein from the transforming growth factor β superfamily [39]. Differential growth factor 15 plays a role in the regulation of inflammation and apoptosis in tissues during damage and during pathological processes. The high content of this protein in the blood is a prognostic factor of heart failure and ACS [40].

When examining the blood of 181 patients with heart failure one month after myocardial infarction in the New Zealand Coronary Disease Cohort Study (CDCS), 212 differentially expressed blood proteins associated with heart failure were found. Of these, 96 proteins correlated with the left ventricular ejection fraction measured four months after MI. The authors identified priority candidate proteins associated with heart failure, including B-Type natriuretic peptide, troponin T, angiopoietin-2, thrombospondin-2, latent growth transforming factor-β-binding protein-4, and follistatin-related protein-3 [41].

Calprotectins are a group of intracellular proteins involved in the growth, reproduction, contraction and differentiation of cells, RNA synthesis, phosphorylation of protein molecules, and the formation of an immune response. Calprotectins can bind calcium, zinc and copper. Some types of calprotectin are tissue-specific. In humans, calprotectin correlates with the degree of coronary and carotid atherosclerosis and with an unstable plaque, it is a marker of myocardial damage. An increased level of calprotectin in blood plasma is associated with an increased risk of coronary events in healthy people and allows us to assess the degree of risk of complications after myocardial infarction [42].

In clinical terms, there is increasing evidence of the role of proteomic technology in the detection of new potential biomarkers. It seems that proteomics will provide new information about the molecular events associated with ACS and potentially lead to the identification of new drug targets. The proteomic approach is very important for identifying new biomarkers related to platelet function and their metabolism in ACS [43,44].

Platelets play a certain role in the vascular system since they are the main mediator of thrombosis. When trying to compare the proteome of intracoronary and peripheral arterial platelets in patients with ST-segment elevation myocardial infarction in search of potential platelet biomarkers, 16 differentially regulated proteins related to cytoskeletal and signalling were identified. The researchers demonstrated an increased regulation of proteins: integrin αIIb, Src kinase-associated phosphoprotein-2 adapter, and thrombospondin-1 isoforms in intracoronary platelets [45].

In the search for new biomarkers and the study of activated platelets, blood proteomes were compared in 13 patients with ACS and 14 patients with stable angina using the tandem mass spectrometry approach. 318 proteins were identified in both cohorts, while nine differentially expressed proteins were detected. At the same time, the analysis revealed three proteins that are either reduced (blood clotting factor 5; fibronectin) or absent (tetranectin) in patients with ST-segment elevation myocardial infarction, which are closely related to the blood clotting cascade [46]. In part, these data are confirmed in a new study of diagnostic markers of ACS using proteomic methods in the blood serum of patients with ACS and a healthy control group. It was shown that the levels of hemopexin, leucine-rich alpha-2 glycoprotein and vitronectin were increased, while the level of fibronectin was lowered in patients with ACS. Thus, the use of these biomarkers can increase the accuracy of the diagnosis of ACS [47]. Investigating the biochemical basis of reverse cholesterol transport using quantitative proteomic methods in patients with clinically diagnosed STeMI, the authors showed that 64 proteins significantly differ between healthy control subjects and subjects with MI. Proteomic analysis revealed a panel of proteins associated with atherosclerosis and MI. One of the proteins, AZGP1 (Zinc-alpha-2-glycoprotein), probably acts as a missing link between chronic inflammation and cholesterol transport. Violation of the reverse cholesterol transport regulation can be controlled by AZGP1, CD36, ABCA5 and PPARɣ in subjects with MI [48].

Proteomic methods using the technology of Isobaric tags for relative and absolute quantitation (iTRAQ) and tandem mass spectrometry made it possible to study the urine proteome to identify potential diagnostic biomarkers of MI. The urine proteome was analysed within 12 h after the appearance of the first symptoms of early myocardial infarction and on the 7th day after percutaneous coronary intervention. In the urine proteome of patients with MI, 12 differentially expressed proteins were associated with atherosclerosis, abnormal coagulation and abnormal cell metabolism, and five proteins (antithrombin-III, complement C3, α-1-acid glycoprotein 1, serotransferrin and cathepsin Z) were first associated with MI. Binary logistic regression analysis showed that a combination of these five urine proteins can be used to diagnose MI with a sensitivity of 94% and a specificity of 93% [49].

## 4. Results of Proteomic Blood Tests for Carotid Artery Atherosclerosis and Stroke

Atherosclerosis is the pathophysiological basis of many diseases. The mass spectrometry study of plaque samples obtained after carotid endarterectomy identified proteins expressed differently when comparing between early and advanced lesions. A total of 95 proteins were overexpressed, and 117 proteins were suppressed in advanced lesions compared to early atherosclerotic lesions (*p* < 0.05). At the same time, activated proteins were associated with proatherogenic processes, while suppressed proteins participated in the organization of the extracellular matrix and the smooth muscle cytoskeleton of blood vessels [50].

Diagnosis of patients with high-risk atherosclerotic plaques before the onset of clinical manifestations remains a difficult task and requires an improved approach to predicting the appearance of symptoms. The proteomic profile of the vascular extracellular matrix and associated molecules in human carotid artery endarterectomy samples from six symptomatic and six asymptomatic patients were compared. Proteomics data were combined with profiling of gene expression of carotid endarterectomy and analysis of protein secretion by lipid-loaded human vascular smooth muscle cells. In patients with symptoms, differentially expressed plaque proteins were identified, such as matrix metalloproteinase 9, calcium-binding protein S100 A8 (S100A8), S100A9, cathepsin B, fibronectin, and galectin-3-binding protein. Biomarker candidates were measured in 685 subjects and were associated with the progression of atherosclerosis. Four biomarkers (matrix metalloproteinase 9, S100A8/S100A9, cathepsin D and galectin-3-binding protein) improved risk prediction in the independent cohort of the SAPHIR study. Thus, the authors concluded that the identified four biomarkers could improve risk prediction and diagnosis for the treatment of cardiovascular diseases [28].

Superoxide dismutase (SOD), together with catalase and other antioxidant enzymes, protects the body from constantly forming highly toxic oxygen radicals. SOD, having the highest known catalytic reaction rate, catalyses the dismutation of the superoxide radical (O_2_^−^) into oxygen and hydrogen peroxide. There are three types of SOD: SOD 1 is located in the cytoplasm, SOD 2 is in the mitochondria, and SOD 3 is an extracellular form. SOD1 and SOD3 contain copper in the active centre and zinc as a structural component, and SOD2 contains manganese in the active centre [51]. The study of protein profiles of blood clots extracted during thrombectomy surgery in 20 patients with acute ischemic stroke with an unclear aetiology of stroke was carried out. The analysis of protein profiles was carried out by the method of nano-liquid chromatography with tandem mass spectrometry. 81 proteins were found in all emboli, five of them (septin 2, phosphoglycerate kinase 1, integrin alpha-M, glucose-6-phosphate dehydrogenase, mitochondrial superoxide dismutase 2 (SOD 2)) positively correlated with high LDL levels [52].

Serum amyloid A (SAA) is a serum protein synthesized by hepatocytes, a marker of the acute phase. A macrophage mediator-interleukin 1 stimulates an increase in the synthesis of SAA in inflammatory diseases. In patients with carotid artery atherosclerosis who underwent carotid endarterectomy, the concentration of SAA protein was increased compared to the proteomic profile of the control group (patients without atherosclerosis). Having compiled a proteomic map of apolipoproteins in different lipoprotein fractions (LDL, HDL), Lepedda and colleagues found that the level of SAA was increased in all lipoprotein fractions in atherosclerosis, but in the LDL fraction, its level was especially high (an increase of 14 times), which shows the connection of this protein with the development of the pathological atherosclerotic process in the carotid arteries [53].

When searching for markers of ischemic stroke, a proteomic analysis was performed in the endothelial progenitor cells of patients with ischemic stroke in comparison with healthy control. Endoplasmic reticulum protein-29 and CdC-42 were expressed only in endothelial progenitor cells of healthy subjects, and elongation factor-2 was identified only in endothelial progenitor cells from patients with ischemic stroke. It was also found that the peroxiredoxin-1 expression in patients with ischemic stroke is 10 times higher than in healthy people [54].

In patients with acute ischemic stroke, four proteins with increased regulation were identified: Insulin-like growth factor 2 (IGF2), lymphatic vessel endothelial hyaluronic acid receptor 1 (LYVE1), the pro-platelet basic protein (PPBP) and thrombospondin 1 compared to healthy controls. These proteins have been independent predictors of a favourable outcome of ischemic stroke [55].

When studying platelets in the pathogenesis of stroke, 83 proteins showed to be differentially expressed in the stroke group, in contrast to the control group. The identified proteins localization in percentage terms is: 36% in the cell’s cytoplasm, 42% in the extracellular space, 11% in the membrane, 3% in the cytoskeleton and 8% localization is unknown. The results showed that several important proteins, such as myeloperoxidase, arachidonate-12-lipoxygenase, histidine-rich glycoprotein, Crk-like protein, vitronectin, thrombospondin 1, apolipoprotein H, apolipoprotein AI, complement component C3, clusterin were present in the platelets of stroke patients. Some suggest that some of these proteins play an important role in platelet activation and the pathophysiology of acute ischemic stroke [56].

## 5. Results of Proteomic Studies of Atherosclerotic Plaques

To study the participation of proteins in the pathological process of coronary atherosclerosis, it is important to study the specific relationships between proteins in the coronary arteries, the expression and concentration of proteins. Temporal analysis of the proteomic profile of the vascular wall in coronary atherosclerosis can help to detect possible diagnostically significant protein structures or potential biomarkers of the disease and develop novel approaches to the diagnosis of coronary atherosclerosis and its complications.

The first large-scale proteomic study of human coronary artery proteins and coronary atherosclerotic plaques identified 806 differentially expressed proteins. The development of atherosclerosis involved some of them, while others may be involved in the disease progression. All of them are made up of four groups: (1) extracellular matrix proteins, (2) lipid-binding proteins and proteins associated with metabolism, (3) proteins associated with inflammation and (4) phagocytic ligands and receptors of apoptotic cells [57].

From the point of view of molecular biology, we can define CHD disease as a community of thousands of proteins that collectively change cellular processes and lead to a characteristic remodelling of the local environment of the coronary artery. To characterize the proteome of human coronary arteries, samples of coronary arteries in 2 autopsy cases (men 64 and 69 years old), divided into 20 segments, were studied using proteomic research methods. 174 differentially expressed proteins were found in pathological and normal intima. The molecular functions of these proteins primarily included: binding (41.47%), catalytic activity (33.24%), carrier activity (9.41%) and structural molecular activity (7.06%) [58].

In the EPICHEART study, when studying the role of epicardial adipose tissue in the pathophysiology of late-stage CHD and the relationship between the volume/proteome of epicardial adipose tissue and progressive coronary atherosclerosis in patients with severe aortic stenosis using SWATH mass spectrometry, it was found that the volume of epicardial adipose tissue was associated with high coronary artery calcium (coronary calcium score), but not with the presence of coronary stenosis. Epicardial adipose tissue demonstrated a procalcifying proteomic profile in patients with coronary heart disease, with increased regulation of annexin A2 and decreased regulation of fetuin-A protein. The level of the protein annexin A2 in the samples of epicardial adipose tissue also positively correlated with high coronary artery calcium, in addition, the annexin A2 gene was overexpressed in the samples of epicardial adipose tissue of patients with coronary heart disease and positively correlated with high coronary artery calcium. The fetuin-A gene was not detected in epicardial adipose tissue samples, but the concentration of fetuin-A protein in the blood was higher in patients with CHD than in patients without CHD [59].

Serum amyloid P-component (SAP) is an acute-phase protein and plays a significant role in the biological processes of the cardiovascular system, such as inflammation and fibrosis. The amount of SAP in the tissues increases with degenerative aortic stenosis. In addition, the levels of SAP in plasma are positively associated with cardiovascular diseases in the elderly [60]. In a study on mouse models with apolipoprotein E deficiency (Apoe −/−), intraperitoneal injection of SAP inhibits atherosclerosis in these animals [61]. Increased SAP expression was observed in haemorrhagic atherosclerotic plaques of the carotid arteries compared to fibrous plaques [62]. Annexin 5 is found in the vascular endothelium and has anti-inflammatory, anticoagulant and antiapoptotic effects due to the binding of phosphatidylserine molecules [63]. It has been shown that the level of annexin 5 in the blood increases significantly after the destruction of the atherosclerotic plaque [64]. In a later proteomic study at the stage of unstable atherosclerotic plaques of the coronary arteries, increased content of SAP and annexin 5 was noted [65].

Using mass spectrometric analysis, Herrington and colleagues studied the human arterial proteome and features associated with early atherosclerosis of coronary arteries and aortic samples (sectional material of 100 young adults, 200 arterial samples). Significant differences were found in the prevalence of mitochondrial protein, tumour necrosis factor α, insulin receptor, PPAR-α and-γ between coronary and aortic samples, between atherosclerotic and normal tissues. It has been shown that some biomarkers of tissue proteins indicating early atherosclerosis predict anatomically defined coronary atherosclerosis, confirming the possibility of using human tissue proteomics for clinical and diagnostic purposes. The authors concluded that the human arterial proteome can be considered as a complex network, the architectural features of which differ significantly depending on the anatomical position and the presence or absence of atherosclerosis [66].

Cyclin-dependent kinases (CDKs) are serine/threonine kinases and phosphorylate the corresponding amino acid residues in proteins. There are 11 cyclin-dependent kinases, each of which is activated by one or more cyclins and other similar molecules after reaching their critical concentration. CDK9 is activated by cyclins T1, T2a, T2b and K. With a decrease in the intracellular concentration of cyclin, reversible CDK inactivation occurs. Han Y. With colleagues found high concentrations of the CDK9 enzyme in patients with coronary atherosclerosis compared to the control group. In addition, high values of the enzyme correlated with a high content of the antigen-like cluster of differentiation 14 and monocytes/macrophages in the atherosclerotic focus. The authors suggest CDK9 may be a potential biomarker of atherosclerotic inflammation [67].

In an earlier study, Lepedda A. J. and colleagues applied proteomic analysis to homogenates of atherosclerotic plaques obtained during endarterectomy in patients with carotid artery atherosclerosis. From the entire mass of proteins, the authors identified a group of 33 proteins that are differentially expressed between stable and unstable plaques. A steady increase in the proteins ferritin, SOD2 and fibrinogen (fragment D) and a decrease in the level of glutathione transferase and SOD3 were found in unstable plaques. Western blot analysis confirmed the data of mass spectrometric analysis. The functional importance of different isoforms of SOD is not yet clear. An increased level of fibrinogen (fragment D) may contribute to the instability of atherosclerotic plaques. In addition, positive correlations were obtained between the level of ferritin in the blood and in the homogenates of atherosclerotic plaques, which allowed the authors to consider ferritin as a potential marker of the progression of atherosclerosis [68].

Similar results were obtained by another group of scientists. When comparing the proteomic profiles of homogenates of stable and unstable atherosclerotic plaques of the same person, it was found that the unstable plaque has a high concentration of ferritin and fibrinogen, and the stable atherosclerotic plaque is dominated by apoE, actin and L-lactate dehydrogenase B. The identified proteins, according to the authors, may be potential markers of complications of atherosclerotic lesions [69].

In the study, Rocchiccioli and colleagues studied 463 proteins isolated from atherosclerotic plaques and blood plasma of patients with atherosclerosis (*n* = 34) who underwent carotid endarterectomy (*n* = 14) and compared them with the protein profile of healthy volunteers. Consistently high levels of thrombospondin-1, a protein that regulates the interaction of cells with each other and with the extracellular matrix, and vitamin D binding protein, were obtained in patients with atherosclerosis. The data were obtained by liquid chromatography and mass spectrometry and confirmed by Western blot analysis [70]. In a study by Malaud et al. using a complex of proteomic research methods, 118 proteins differentially expressed in fibrous and haemorrhagic plaques were detected. This allowed the authors to identify three biological processes associated with atherosclerosis (platelet degranulation, vascular autophagy and negative regulation of fibrinolysis). The data of proteomic studies made it possible to identify new biomarkers (calponin-1, DJ-1, vascular endothelial growth factor (VEGF) and Procollagen C-proteinase enhancer-1 (PCPE-1)) of plaque vulnerability, which increased the importance of proteolysis, SMC and extracellular matrix integrity, inflammation, oxidative stress, platelet degranulation and autophagy in the progression of atherosclerotic foci [62].

In the study of cancer biomarkers, it was found that vascular smooth muscle cells (VSMC) have a different and unusual morphology in the atherosclerotic plaque, which correlates with the proliferative state of the cells. Proteomic analysis reveals proteins associated with the formation of atherosclerosis, including mimecan (osteoglycin), Ras-1 suppressor protein (RSUP-1) and cathepsin D, which are simultaneously identified as biomarkers of cancer tumours. The expression of mimecan and RSUP-1 is suppressed in the atherosclerotic plaque, while the expression of cathepsin D is increased [71]. Earlier studies also identified a decrease in the expression of osteoglycin in haemorrhagic atherosclerotic plaques, which, according to the authors, can lead to plaque instability [62]. On the other hand, there are studies with the opposite point of view. It has been shown that the amount of osteoglycin in the blood of patients with coronary heart disease increases, but does not correlate with the severity of the disease. However, in patients with complex coronary lesions, its level was reduced, osteoglycin plays a role in stabilizing coronary plaques [72]. In a study that of the prognostic value of certain biomarker proteins for patients with coronary heart disease, circulating osteoglycin (mimecan), whose expression is increased in vulnerable atherosclerotic plaques, was called a promising biomarker of adverse cardiovascular events [73]. A study performed using proteomic methods confirmed the high content of mimecan in samples of stable fibrous and unstable necrotic-dystrophic atherosclerotic plaques in patients with coronary atherosclerosis [65].

Endothelial cells form a metabolically active barrier between the vascular lumen and the vascular wall. Oxidative stress and modifications of tubulin, a component of endothelial cell microtubules, destabilize the integrity of blood vessels and increase permeability, which leads to an increase in cardiovascular risk [74]. It is known about the high expression of cytokeratins inside progressive atherosclerotic foci at the level of mRNA and protein [75]. In rabbits with hyperlipidaemia and atherosclerotic changes, regulating tropomyosin, actin and keratin proteins in the tissues of the carotid artery and the middle cerebral artery are increased [76]. Mutations in the tropomyosin 1 gene can cause hereditary cardiomyopathies, left ventricular hypertrophy, or diastolic function disorders in the absence of hypertension and aortic stenosis [77].

In patients with coronary atherosclerosis, the proteomic profile of stable atherosclerotic plaques of the coronary arteries showed a significant increase in the content of proteins: actin, tropomyosin, vimentin, keratin, tubulin and microfibrils of associated glycoprotein 4 (MAGP-4) [65].

Human serum albumin (HSA) is the main protein of human blood plasma. It has been demonstrated that a low concentration of HSA in the blood is a prognostic factor of atherosclerosis in blood vessels, regardless of traditional risk factors in patients with HIV infection. In addition, HSA was associated with markers of systemic inflammation and hypercoagulation (interleukin 6, tumour necrosis factor α, C-reactive protein, fibrinogen and D-dimer). The pathophysiological mechanism underlying this association is the ability of HSA to bind many ligands, including proatherogenic ones, preventing their contribution to oxidative stress [78]. Unstable atherosclerotic plaque is characterized by overexpression of various proatherogenic factors and ligands, which may lead to the transfer of HSA from blood plasma to atherosclerotic foci. A proteomic study of unstable atherosclerotic plaque of the necrotic-dystrophic type of coronary arteries confirmed an increased content of HSA and fibrinogen [65].

## 6. Conclusions

In this review, based on the literature data, we tried to show the range of proteomic studies that can become the basis for the use of certain proteins as potential biomarkers for early diagnosis, prognosis and a better understanding of the pathophysiology of CVD.

Summary data on various markers of the atherosclerotic process are presented in Table 1.

In conclusion, it should be noted that at the last annual international congresses on proteomics (World Congress of the Human Proteome Organisation (https://hupo2021.org/wp-content/uploads/2021/11/HUPO-2021-Abstract-Book_Oct-27.pdf); 9th International Conference and Expo on Proteomics (https://www.aconf.org/conf_101120.html); 89 EAS Congress (Atherosclerosis, 2021, 331, e102. https://doi.org/10.1016/j.atherosclerosis.2021.06.301), et al.), priority results of a meta-analysis of the conducted studies on the search and determination of new proteomic biomarkers for early effective diagnosis and risk assessment of the development of CVD are reported. Information is also announced about such studies currently being conducted, but not yet completed, in atherosclerotic CVD. The results of studies conducted by European and American scientists in recent years indicate the high prospects and extreme relevance of proteomic studies, including tissue proteomic studies in atherosclerosis. New proteomic biomarkers may have informational value in addition to biomarkers already used in clinical practice to improve early diagnosis, treatment and prediction of the risk of cardiovascular diseases.

## Figures and Tables

**Table 1:** Proteomic biomolecules associated with atherosclerosis and its complications.

	Proteomic Biomolecules	References
Elevated blood levels in patients with coronary atherosclerosis and coronary heart disease	Growth differentiation factor 15 Proprotein convertase of subtilisin-kexin type 9 Transferrin receptor protein 1 Galectin-3 Galectin-4 Interleukin 17 Calprotectin Cathepsin D Complement components C3 (Chain B), C4, C9 Matrix metalloproteinase 9 Renin Sialylated glycans NO synthase C-reactive protein the Willebrand factor vascular endothelial growth factor	Alexandar, V. et al. [14] Krishnan, S. et al. [15] Stakhneva, E.M. et al. [18] Ljungberg, J. et al. [27] Langley, S.R. et al. [28] Ferrannini, G. et al. [31]
Elevated in the blood in patients with in ACS and MI	Zinc-alpha-2-glycoprotein Cluster of differentiation 5 α-1-acid glycoprotein 1 Antigen-like differentiation cluster 5 (CD5) Leucine-rich alpha-2 glycoprotein Vitronectin Haptoglobin Hemopexin Growth differentiation factor 15 Calprotectin Cell-surface glycoprotein mucin cell surface-associated protein 18 Multimerin-2 Paraoxanase 1 Polygenic immunoglobulin receptor Salivary α-amylase 1 Tetranectin Complement factor C5a Collagen chain-1 Cyclophilin A Cystatin C	de Hoog, V.C. et al. [35] Yin, X. et al. [36] Mohamed Bakrim, N. et al. [38] Xu, X. et al. [40] Schiopu, A. et al. [42] Shin M. et al. [47] Das A. A. et al. [48]
Elevated in the blood in patients with carotid artery atherosclerosis and stroke	Crk-like protein S100A8/S100A9 Apolipoprotein AI Apolipoprotein H Arachidonate-12-lipoxygenase Vitronectin Galectin-3-binding protein Histidine-rich glycoprotein Glucose-6-phosphate dehydrogenase Growth hormone Growth differentiation factor 15 Insulin-like growth factor 2 Integrin alpha-M Cathepsin D Clusterin Complement component C3 Matrix metalloproteinase 9 Matrix metalloproteinase 12 Myeloperoxidase Mitochondrial superoxide dismutase 2 Pro-platelet basic protein Osteoprotegerin Peroxiredoxin-1 Renin Lymphatic vessel endothelial hyaluronic acid receptor 1 Septin 2 Serum Amyloid A (SAA) T-cell immunoglobulin and mucin domain 1 Thrombospondin 1 Elongation factor-2 Phosphoglycerate kinase 1 member of the tumour necrosis factor superfamily 14	Langley, S.R. et al. [28] Lind L. et al. [29] Rao, N.M. et al. [52] Lepedda A. J. et al. [53] Brea D.et al. [54] Qin C. et al. [55] Cevik O. et al. [56]
Elevated in unstable atherosclerotic plaques	Protein Deglycase DJ-1 Annexin 5 Vitamin D binding protein Calponin-1 Cathepsin D Mimekan Superoxide dismutase 2 Serum Amyloid P-component Thrombospondin 1 Procollagen C-proteinase enhancer-1 Vascular endothelial Growth Factor Ferritin Fibrinogen Cyclin-dependent kinases Human Serum Albumin	Malaud E. et al. [62] Stöhr R. et al. [63] Lee R. et al. [64] Stakhneva E. M. et al. [65] Han Y. et al. [67] Lepedda A. J. et al. [68] Olson F.J. et al. [69] Rocchiccioli S. et al. [70] Fasehee H. et al. [71] Cheng J.M. et al. [73]

## Data Availability

Not applicable.
